# Molecular diagnosis to discriminate pathogen and apathogen species of the hybrid *Verticillium longisporum* on the oilseed crop *Brassica napus*

**DOI:** 10.1007/s00253-012-4530-1

**Published:** 2012-11-16

**Authors:** Van Tuan Tran, Susanna A. Braus-Stromeyer, Christian Timpner, Gerhard H. Braus

**Affiliations:** Institut für Mikrobiologie und Genetik, Georg-August-Universität Göttingen, Grisebachstr 8, 37077 Göttingen, Germany

**Keywords:** *Verticillium*, Interspecies hybrid, rDNA homogenization, Regulatory isogene pairs

## Abstract

The cruciferous fungal pathogen *Verticillium longisporum* represents an allodiploid hybrid with long spores and almost double the amount of nuclear DNA compared to other *Verticillium* species. *V*. *longisporum* evolved at least three times by hybridization. In Europe, virulent A1xD1 and avirulent A1xD3 hybrids were isolated from the oilseed crop *Brassica napus*. Parental A1 or D1 species are yet unknown whereas the D3 lineage represents *Verticillium dahliae*. Eleven *V*. *longisporum* isolates from Europe or California corresponding to hybrids A1xD1 or A1xD3 were compared. A single characteristic type of nuclear ribosomal DNA could be assigned to each hybrid lineage. The two avirulent A1xD3 isolates carried exclusively D3 ribosomal DNA (rDNA) which corresponds to *V*. *dahliae*. The rDNA of all nine A1xD1 isolates is identical but distinct from D3 and presumably originates from A1. Both hybrid lineages carry two distinct isogene pairs of four conserved regulatory genes corresponding to either A1 or D1/D3. D1 and D3 paralogues differ in several single nucleotide polymorphisms. Southern hybridization patterns confirmed differences between the A1 and D1/D3 isogenes and resulted in similar patterns for D1 and D3. Distinct signatures of the *Verticillium* transcription activator (*VTA*)*2* regulatory isogene pair allow identification of *V*. *longisporum* hybrids by a single polymerase chain reaction and the separation from haploid species as *V*. *dahliae* or *Verticillium albo*-*atrum*. The combination between *VTA2* signature and rDNA type identification represents an attractive diagnostic tool to discriminate allodiploid from haploid *Verticillia* and to distinguish between A1xD1 and A1xD3 hybrids which differ in their virulence towards *B. napus*.

**Electronic supplementary material:**

The online version of this article (doi:10.1007/s00253-012-4530-1) contains supplementary material, which is available to authorized users.

## Introduction

Speciation is a dynamic process, which requires the isolation of different populations to prevent the exchange of genetic material. Reproductive barriers are formed by changes in ploidy, chromosomal rearrangements or incompatibilities between genes. Reproductive barriers are prerequisites for the evolution of diverging genetic elements, which convey an ecological separation (Mallet 2007; Giraud et al. 2010).

The fungal genus *Verticillium* has a world-wide distribution and includes species which cause vascular wilting disease and early senescence in a broad range of economically important crops including alfalfa, lettuce, hops, olive trees, oilseed rape, potato, tomato or strawberries (Pegg and Brady 2002). *Verticillium* morphology includes a characteristic verticilliate arrangement of the three to five asexual spore producing cells (phialides) forming branches at each node of the conidiophores (Kim et al. 2001). *Verticillium dahliae* and *Verticillium albo*-*atrum* are two closely related but distinct mature species. *V*. *albo*-*atrum* forms melanized resting mycelium, whereas the *V*. *dahliae* hyphae are not black. Instead, *V*. *dahliae* forms resting black microsclerotia, which are melanized clumps formed by budding of mycelial cells (Reinke and Berthold 1879; Isaac 1967; Goud et al. 2003). There is an overlap in host specificity, but both fungi can be distinguished by infecting different cultivars of a host plant species (Kim et al. 2001; Zeise and von Tiedemann 2002a). *V*. *dahliae* has a significantly broader host range and is able to infect more than 200 plant species. *V*. *albo*-*atrum* has in addition a specific host adaptation to lucerne and other leguminoses or to hops from the cannabaceae family (Pegg and Brady 2002; Agrios 2005; Vallad et al. 2005; Klosterman et al. 2009).

The list of hosts infected by *Verticillium* species is expanding and there is a continuous increase in severity of disease outbreaks on known hosts (Pegg and Brady 2002; Inderbitzin et al. 2011). Therefore, *Verticillium* represents an interesting and relevant model to study speciation by comparing nascent to already significantly diverged mature species. Crucifers are hardly infected by *V*. *dahliae* or *V*. *albo*-*atrum*. However, in 1961 a *Verticillium* was isolated from wilted horseradish as a first member of the crucifer family of *Brassicaceae* (Stark 1961). The isolated fungus forms preferentially only three phialides per node and survives by means of black microsclerotia which are elongated when compared to *V*. *dahliae* (Karapapa et al. 1997). The name *Verticillium longisporum* refers to twice (7.1–8.8 μm) as long asexual spores (conidia) in comparison to *V*. *dahliae* (3.5–5.5 μm; Karapapa et al. 1997; Zeise and von Tiedemann 2001; Collins et al. 2003). The numbers of *V*. *longisporum* isolates from crucifers are increasing and include horseradish from Illinois (Eastburn and Chang 1994), oilseed crops from Europe and Canada (Heale and Karapapa 1999; Zeise and von Tiedemann 2001; Steventon et al. 2002; Gladders et al. 2011) or cauliflower from California (Koike et al. 1994).

*V*. *longisporum* isolates are “near-diploid” or amphihaploid fungi with higher nuclear DNA amounts (about 1.8 times) than those of *V*. *dahliae* or *V*. *albo*-*atrum* isolates (Karapapa et al. 1997; Steventon et al. 2002; Collins et al. 2003). This almost diploid status might be the reason why numerous mutagenesis approaches have failed (Ingram 1968; Hastie 1973; Jackson and Heale 1985; Nagao et al. 1994; Subbarao et al. 1995; Zeise and von Tiedemann 2001). Most filamentous ascomycetes are haploid (Glass and Kuldau 1992). Experimental studies with the model fungus *Aspergillus nidulans* suggest that during adaptation to a novel environment, haploids deriving from diploids by parasexual recombination reach a higher fitness than the original diploids (Schoustra et al. 2007). Short-spored crucifer isolates might be haploid recombinants of long-spored isolates and *V*. *longisporum* distinct from noncrucifer isolates of *V*. *dahliae* (Karapapa et al. 1997; Barbara and Clewes 2003; Collins et al. 2003; Qin et al. 2006; Clewes et al. 2008).

Amplified fragment length polymorphism and restriction fragment length polymorphism originally suggested *V*. *longisporum* as interspecies hybrid between *V*. *dahliae* and *V*. *albo*-*atrum* or of close relatives of these two species (Karapapa et al. 1997; Steventon et al. 2002; Collins et al. 2003; Fahleson et al. 2004; Clewes et al. 2008; Collado-Romero et al. 2010). A recent comparison of eight genetic loci including the ribosomal internal transcribed spacer (ITS) regions and genes for two structural proteins (actin, tubulin), one transporter (mitochondrial oxalacetate transport protein), one translation factor (EF1-α), two enzymes (glyceraldehyde-3-P-dehydrogenase, tryptophan synthase) and the genes for the mating types (MAT1-1) suggested that *V*. *longisporum* hybridized at least three times resulting in the lineages A1xD1, A1xD2, A1xD3, with A1xD2 exclusively found in the USA. The A1 and D1 had been described as yet unknown *Verticillium* species, whereas D2 and D3 represent *V*. *dahliae* lineages (Inderbitzin et al. 2011).

In this work, we compared *V*. *longisporum* A1xD1 hybrids from Europe and California which are virulent for oilseed rape and cauliflower, respectively, with avirulent A1xD3 isolates from European oilseed rape. We focused on rDNA and four regulatory gene pairs to identify molecular differences on a genomic level which allow an accurate molecular diagnosis to discriminate allodiploid hybrids from each other and from haploid *Verticillium* relatives.

## Materials and methods

### Fungal isolates and maintenance

Twenty-six isolates of *Verticillium* species from different hosts were used in this study (Table 1). For the long-term maintenance, the isolates were grown separately in liquid Czapek-Dox or simulated xylem medium (SXM; Neumann and Dobinson 2003) for 10 days at 25 °C. Conidia were harvested by filtering the culture through Miracloth (Calbiochem, Darmstadt, Germany) the filtrate was centrifuged at 5,000 rpm for 10 min. After a washing step with sterile water, the pellet was resuspended in sterile water and the number of spores was counted under a microscope. Glycerin was added to the spore suspension at the final concentration of 20 % and aliquots of the spore suspension were frozen in liquid nitrogen and stored at −80 °C.

**Table 1:** *Verticillium* strains used in this study

Name	Species	Lineage	Virulence on plants	Original plant host	Geographical origin
Vl18	*V*. *longisporum*	A1xD1	+	*B. napus* (oilseed rape)	Mecklenburg/Germany^a^
Vl40	*V*. *longisporum*	A1xD1	+	*B. napus* (oilseed rape)	Mecklenburg/Germany^a^
Vl43	*V*. *longisporum*	A1xD1	+	*B. napus* (oilseed rape)	Mecklenburg/Germany^a^
Vl59	*V*. *longisporum*	A1xD1	+	*Brassica oleracea* (cauliflower)	California/USA^a^
Vl60	*V*. *longisporum*	A1xD1	+	*Brassica oleracea* (cauliflower)	California/USA^a^
Bob70	*V*. *longisporum*	A1xD1	+	*Brassica oleracea* (cauliflower)	California/USA^b^
Vl82	*V*. *longisporum*	A1xD1	+	*B. napus* (oilseed rape)	Mecklenburg/Germany^a^
Vl83	*V*. *longisporum*	A1xD1	+	*B. napus* (oilseed rape)	Mecklenburg/Germany^a^
Vl84	*V*. *longisporum*	A1xD1	+	*B. napus* (oilseed rape)	Mecklenburg/Germany^a^
Vl19	*V*. *longisporum*	A1xD3	−	*B. napus* (oilseed rape)	Mecklenburg/Germany^a^
Vl32	*V*. *longisporum*	A1xD3	−	*B. napus* (oilseed rape)	Mecklenburg/Germany^a^
Vd2	*V*. *dahliae*		+	*Fragaria x ananassa* (strawberry)	Münsterland/Germany^a^
Vd8	*V*. *dahliae*		+	*Solanum tuberosum* (potato)	Münsterland/Germany^a^
Vd13	*V*. *dahliae*		+	*Gossypium hirsutum* (cotton)	Cordoba/Spain^a^
Vd39	*V*. *dahliae*		+	*Helianthus annuus* (sunflower)	Hessen/Germany^a^
Vd52	*V*. *dahliae*		+	*Capsicum annuum* (chili pepper)	Burgenland/Austria^a^
JR2	*V*. *dahliae*		+	*Solanum lycopersicum* (tomato)	Canada^c^
Vd14.01	*V*. *dahliae*		+	*Pistacia vera* (pistachio)	USA^c^
Vd73	*V*. *dahliae*		+	*Linum usitatissimum* (linseed)	Mecklenburg/Germany^a^
Vd89	*V*. *dahliae*		+	*Lupinus luteus* (pea)	Mecklenburg/Germany^a^
Va1	*V*. *albo*-*atrum*		nd	*Solanum tuberosum* (potato)	USA^a^
Va2 (CBS 453.51)	*V*. *albo*-*atrum*		nd	*Medicago sativa* (alfalfa)	UK^d^
Va3 (CBS 393.91)	*V*. *albo*-*atrum*		nd	*Humulus lupulus* (hop)	Belgium^d^
Va4 (CBS 322.91)	*V*. *albo*-*atrum*		nd	*Solanum lycopersicum* (tomato)	Netherlands^d^
Vtr1 (CBS101220)	*V*. *tricorpus*		nd	*Brassica*	Israel^d^
Vni1 (CBS 175.75)	*V*. *nigrescens*		nd	*Solanum tuberosum*	Germany^d^

(*+*) virulent, (*−*) significantly avirulent, *nd* not determined

^a^Zeise and von Tiedemann (2001, 2002a, b)

^b^Qin et al. (2006)

^c^de Jonge et al. (2012)

^d^CBS culture collections of micro-organisms, Utrecht, The Netherlands

### Isolation of nucleic acids

The isolates were grown for one week, at 25 °C in potato dextrose broth (Sigma-Aldrich Chemie GmbH, Munich, Germany) for genomic DNA or liquid SXM (Neumann and Dobinson 2003) for total RNA extraction. The fungal mycelia harvested with Miracloth (Calbiochem, Darmstadt, Germany) were ground to fine powder in liquid nitrogen. The fungal powder was used directly for nucleic acid extraction or stored at −80 °C for later use. Genomic DNA was extracted from the fungal powder (Kolar et al. 1988). Total RNA was extracted by using the RNeasy Plant Mini kit (Qiagen, Hilden, Germany) according to the manufacturer’s instructions. Total cDNA was generated from the total RNA by using RevertAid First-Strand cDNA synthesis kit (Fermentas, St. Leon-Rot, Germany).

### PCR amplification, cloning and sequencing

Polymerase chain reaction (PCR) amplifications were performed in 25 μl volumes with the high-fidelity Phusion polymerase (Finnzymes, Finland) using the following general conditions: an initial denaturation at 98 °C for 3 min; a cycle including denaturation at 98 °C for 30 s, annealing at 55–60 °C for 40 s, and extension at 72 °C for 1–4.5 min was repeated for 25–30 times; a final extension at 72 °C for 7 min and stored at 4 °C until used. PCR products were analyzed on a 1 % agarose gel and the DNA fragments were excised and purified with QIAquick gel extraction kit (Qiagen, Hilden, Germany). The purified DNA fragments were used for direct sequencing. For cloning, each DNA fragment of interest was amplified from *V*. *longisporum* genomic DNA by the high-fidelity Phusion polymerase with modifications optimized for interspecies hybrid microorganisms including increases in concentration of each primer (100 pmol) and each dNTP (20 mM) for each reaction of 25 μl, an increase in time for elongation step (4.5 min) together with a decrease in number of PCR cycles (25 cycles; Beser et al. 2007; Inderbitzin et al. 2011). The PCR product was ligated into the pJET1.2/blunt cloning vector using the CloneJET™ PCR Cloning Kit (Fermentas, St. Leon-Rot, Germany). The ligation mixture was transformed into *E*. *coli* DH5α competent cells. Colony PCR with Hot-Star Taq MasterMix (Qiagen, Hilden, Germany) was used to screen positive colonies from each cloning procedure. At least, 10–15 positive clones were selected and grown in LB (Luria-Bertani) liquid medium containing 100 μg/ml of ampicillin. Recombinant plasmids were purified with the QIAprep Spin Miniprep Kit (Qiagen, Hilden, Germany) for sequencing. The sequencing was performed with the primers (forward and reverse) from the cloning kit by the in-house Göttingen Genomics Laboratory. DNA alignments with the Geneious Pro v5.4 software (Drummond et al. 2011) were performed to detect artificially chimeric sequences generated by PCR as by-products for elimination.

### Southern hybridization

The target genes contain no restriction sites for the enzymes used for genomic DNA digestion. A fragment (about 600 bp) of the target gene or the whole gene was amplified and labeled as probe for Southern hybridization. In brief, 20 μg of genomic DNA were digested with 3 μl of an appropriate restriction enzyme for overnight. The digested mixture was analyzed on a 1 % agarose gel, and DNA was transferred to an Amersham Hybond-N membrane (GE Healthcare, Munich, Germany) by blotting. The DNA on the membrane was overnight hybridized to a specific probe and the Amersham CDP-Star Detection reagent (GE Healthcare, Munich, Germany) was used to detect chemiluminescence signals according to the manufacturer’s instructions.

### DNA analysis

Characterization of 18S rRNA gene, ITS1, 5.8S rRNA, ITS2, 28S rRNA, and intergenic region (IGS) of the rDNA was based on Genbank accession number AF104926 (Pramateftaki et al. 2000). For the other conserved genes, the introns and exons were determined by comparing the cDNA sequences with their genomic DNA sequences using the ClustalW (Thompson et al. 2002). Detection of repeated signatures in the DNA sequences was carried out with Repeats Finder implemented in the multiplatform open-source software Unipro UGENE v1.10 (Okonechnikov et al. 2012). For DNA analysis and comparison in more details, the commercial software Geneious Pro v5.4 (Drummond et al. 2011) was used. Phylogenetic trees were constructed with the MEGA 5.0 software (Tamura et al. 2011) based on the neighbor-joining method (Saitou and Nei 1987). Statistical reliabilities of the internal branches were assessed for the trees with bootstraps of 1,000 replicates. The *p* distance model was used and gaps/missing data were treated with complete deletion or pairwise deletion.

### EMBL accessions

DNA sequences were deposited in the EMBL-Bank with the accession numbers HE972012–HE972156.

## Results

### rDNA of *V. longisporum* isolates is homogenized to D3 rDNA for avirulent and A1 rDNA for virulent lineages

**Fig. 1:**
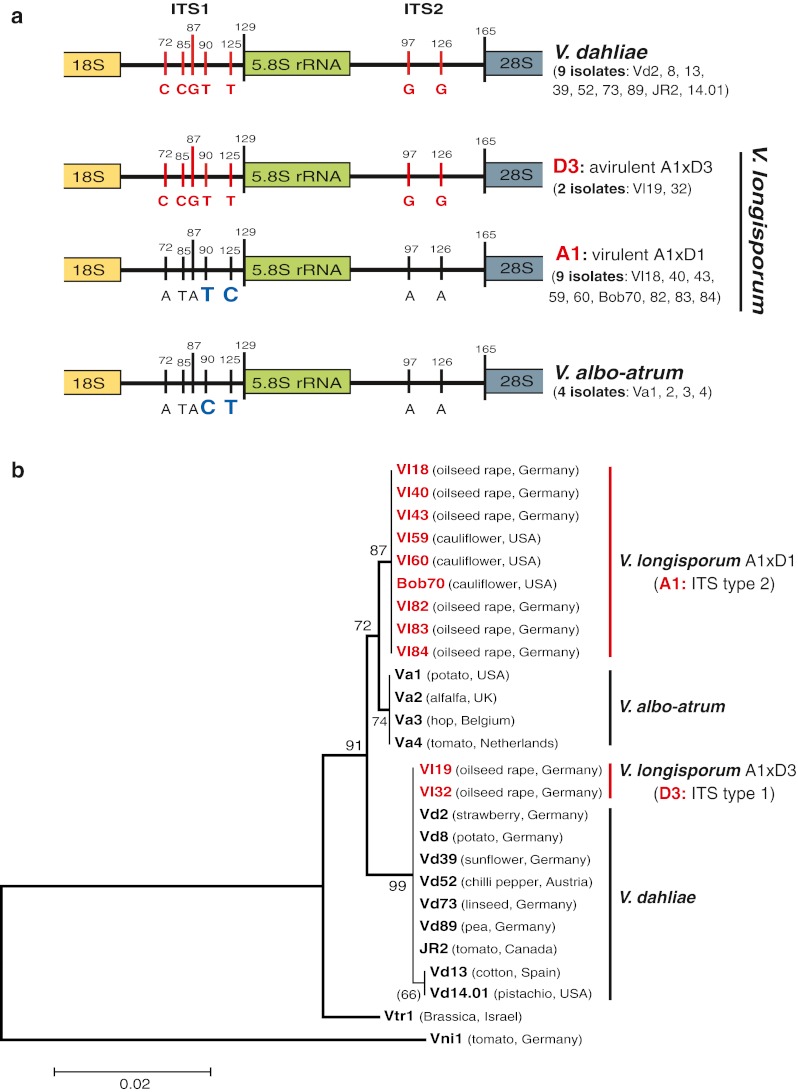
Analysis of ITS region of ribosomal DNA in *Verticillium* plant pathogens. **a** *V*. *longisporum* isolates carry only one type of ITS. The *V*. *dahliae* D3 type corresponds to nine *V*. *dahliae* isolates. The A1 type corresponding to the unknown species A1 is closer related to *V*. *albo*-*atrum* than to *V*. *dahliae*. The two pyrimidine exchanges between ITS1 of A1 and *V*. *albo*-*atrum* at positions 90 and 125 are indicated in *bold*. The ITS region comprises ITS1 (129 nucleotides), 5.8S rRNA and ITS2 (165 nucleotides). A1xD3 strains (Vl19 and Vl32) isolated from oilseed rape in Northern Germany are avirulent, whereas the A1xD1 strains from oilseed rape in Europe or from cauliflower in California are virulent. **b** ITS-based phylogenetic analysis. Avirulent A1xD3 isolates carry *V*. *dahliae* ITS, whereas virulent A1xD1 isolates carry A1 ITS closer related to *V*. *albo*-*atrum* strains. The tree includes two additionally analyzed *Verticillia* for comparison: *V*. *tricorpus* is distant from *V*. *longisporum*, *V*. *dahliae*, and *V*. *albo*-*atrum*; *V*. *nigrescens* Vni1 (now *Gibellulopsis nigrescens*) represents the root of the phylogenetic tree

Allodiploid *V*. *longisporum* species were suggested to be interspecific hybrids (Karapapa et al. 1997; Steventon et al. 2002; Collins et al. 2003; Fahleson et al. 2004; Clewes et al. 2008; Collado-Romero et al. 2010). We have analyzed and compared the rDNA from 24 *Verticillium* isolates of three species including 11 *V*. *longisporum*, 9 *V*. *dahliae*, and 4 *V*. *albo*-*atrum* that originated from different hosts of different geographical regions (Table 1) for a more comprehensive picture of the evolution of *V*. *longisporum* (Fig. 1). Parts of rDNA are commonly used as taxonomic markers (Schoch et al. 2012). The copy numbers of the individual rDNAs vary between 30 and 30,000 copies in eukaryotes (Rooney and Ward 2005; Ganley and Kobayashi 2007).

**Fig. 2:**
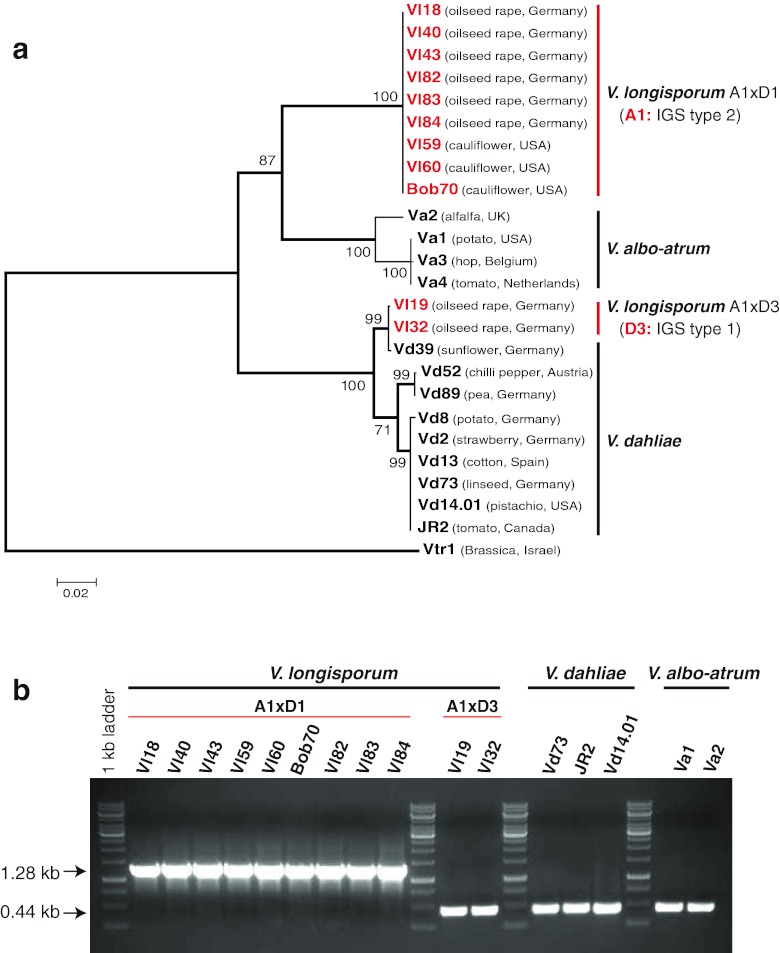
Analysis of the rDNA IGS located between 28S rRNA and 18S rRNA gene of *Verticillium* isolates. **a** IGS sequences of indicated strains which vary in their sequences between 1.7 and 1.9 kb (Fig. S1) were compared by a phylogenetic tree. The IGS sequence of *V*. *tricorpus* distant from all three species is used as root. A1 IGS rDNA (from A1xD1) group is closer to *V*. *albo*-*atrum* rDNA, whereas the D3 IGS (from A1xD3) is the closest to *V*. *dahliae* Vd39 isolated from sunflower. **b** A1 18S rRNA length of virulent A1xD1 isolates was compared to the 18S rRNA of the other strains. A1 18S rRNA carries an additional intron of 839 nucleotides which is as well absent in 18S rRNA of D3 of avirulent A1xD3 isolates as in 18S rRNA of *V*. *dahliae* or *V*. *albo*-*atrum* isolates

Ribosomal DNA repeats including 18S rRNA gene, two internal transcribed spacers (ITS1, ITS2) covering 5.8S rRNA gene and a large IGS were analyzed (Figs. 1 and 2). PCR amplification using various primer pairs ITS-F/ITS-R and IGS-F/IGS-R (Table 2) resulted for each primer pair in only one single sequence.

**Table 2:** PCR primers used in this study

Primer	Sequence (5′–3′)	Target DNA sequence	Product size (bp)	Reference
ITS-F	AGTAAGCGCAAGTCATCAGC	ITS1–5.8S–ITS2	850	AF104926^a^
ITS-R	AAGGAACCATAACTCGAAGCAT			
IGS-F	ACGATCTGCTGAGGGTAAGC	IGS region of rDNA	1,700–1,900	AF104926^a^
IGS-R	ATTCGCAGTTTCGCTTTGTAA			
18S-rRNA1	GGGGATCGAAGACGATCAG	18S rRNA gene	440–1,280	AF104926^a^
18S-rRNA2	TATTGCCTCAAACTTCCATCG			
*VEL1*-F	ATGTCCGCCACCACCAT	*Velvet*-like gene 1 (*VEL1*)	612–1,718	This study^b^
*VEL1*-612-R	TCATGCGGAGGTAGAATCC			
*VEL1*-R	TCATTTTGTGAAAATAGGCGTGTA			
*VEL2*-F	ATGAGCTACGACCAGCACC	*Velvet*-like gene 2 (*VEL2*)	1,620	This study^b^
*VEL2*-R	CTAATAATCGTCATCGTCGTCAT			
*VTA1*-F	ATGTCTTCAAGTTCCAAGACCC	*VTA1* gene	1,275–1,278	This study
*VTA1*-R	TCAGGCACGTTTCATTCCAC			
*VTA2*-F	ATGTACCTGGTCCCCACGCAGC	*VTA2* gene	1,701–1,759	This study
*VTA2*-R	CTAGTGGCCCTGCCCAGGCT			
*sVTA2*-F	GCACGTCACCATGCAGTC	*VTA2* gene-specific	315–383	This study
*sVTA2*-R	CAGCTTCTTCCTCCTTCTTGC			

^a^The primers were designed in this study based on the accession number AF104926 for *Verticillium* rDNA (Pramateftaki et al. 2000)

^b^The primers were designed in this study based on the loci for *VEL1* (VDAG_06763, VDBG_07119) and for *VEL2* gene (VDAG_08715, VDBG_04492) from the BROAD *Verticillium* group Database (Klosterman et al. 2011)

The ITS-F and ITS-R primers amplified DNA includes a part of the 18S rRNA gene (212 bp), ITS1 sequence (129 bp), 5.8S rRNA gene (158 bp), ITS2 sequence (165 bp), and a part of 28S rRNA (178 bp). Sequence analyses revealed that the 5.8S rRNA gene located between ITS1 and ITS2 is completely conserved within all three species. All nine *V*. *dahliae* isolates are separated from all four *V*. *albo*-*atrum* isolates in the ITS1 and the ITS2 region by characteristic nucleotide polymorphisms (Fig. 1a).

There are two different ITS types (ITS type 1 and ITS type 2) within the 11 *V*. *longisporum* isolates. The ITS type 1 of the two European isolates Vl19 and Vl32 is identical to the ITS of *V*. *dahliae*. Vl19 and Vl32 have been isolated from oilseed rape in Europe and are both avirulent (Zeise and von Tiedemann 2002a, b). European *V*. *longisporum* isolates represent either the A1xD1 or A1xD3 hybrid lineages with D3 being the only parental strain which corresponds to *V*. *dahliae* (Inderbitzin et al. 2011). This suggests that Vl19 and Vl32 represent A1xD3 hybrids. The ITS type 2 is close to the ITS of *V*. *albo*-*atrum* except for two pyrimidine exchanges in the ITS1 (Fig. 1a). The ITS type 2 was detected for six virulent European isolates (Vl18, Vl40, Vl43, Vl82, Vl83, Vl84) from oilseed rape (Zeise and von Tiedemann 2002a) and three American virulent cauliflower isolates (Vl59, Vl60, Bob70; Zeise and von Tiedemann 2002a, b; Qin et al. 2006). A1 and D1 are unknown species which are distinct from *V*. *dahliae* or from *V*. *albo*-*atrum*. A1 has been described to be closer related to *V*. *albo*-*atrum* than to *V*. *dahliae*, which is supported by the phylogenetic tree analysis (Fig. 1b). Therefore the ITS type 2 was assigned as the ITS of the unknown *V*. *albo*-*atrum* related species A1.

A closer inspection of the IGS separating rDNA repeats revealed PCR products ranging from 1.7 to 1.9 kb using the primer pair IGS-F/IGS-R. The IGS region is highly polymorphic within all three species and can be divided into two parts based on the sequence conservation (Fig. S1). The variable part of approximately 1 kb starts at the end of the 28S rRNA gene and contains sequences which can be repeated and numerous single nucleotide polymorphisms. In contrast, the conserved part of about 0.7–0.9 kb is situated adjacent to the 18S rRNA gene and reveals high similarity within all three species. All results are summarized in a phylogenetic tree (Fig. 2a).

In agreement to the data obtained with the ITS, there are two different IGS sequence types (IGS types 1 and 2) within the 11 *V*. *longisporum* isolates. The IGS type 1 (two European isolates Vl19 and Vl32) groups within the *V*. *dahliae* clade and corresponds to D3 (Fig. 2a). It contains a single signature of 81 nucleotides (AGCTACCCGGGAATTGGACCAGTTTTGAGGCGGCAGCTACCCGGGAGTTGGCGAAAAACGACCAAGTCGGACACCTTGG). The single copy of the 81-nucleotide signature of the D3 IGS of A1xD3 hybrids corresponds to the IGS of *V*. *dahliae* Vd39 from sunflower, whereas there are two or three repeats of this sequence in the IGS of the other analyzed *V*. *dahliae* isolates (Fig. S1).

The IGS type 2 of the A1xD1 isolates is closer to the clade of the IGS region of *V*. *albo*-*atrum* and corresponds to A1 (Fig. 2a). The A1 IGS of the virulent *V*. *longisporum* A1xD1 lineages includes three copies of a 39 nucleotide repeat (ATCTGGGAGCTACCCGGGAGTTGGA AATTTGGAGAACGG) in the IGS region from European oilseed rape and four repeat copies of the same sequence in the IGS of the American A1xD1 isolates from cauliflower. *V*. *albo*-*atrum* IGS differs from A1 IGS and has no specific repeats in the IGS region of rDNA (Fig. S1).

We aimed to discriminate between the two *V*. *longisporum* hybrids A1xD1 (virulent) and A1xD3 (avirulent) by a single PCR reaction. The 18S rRNA gene-specific primer pair (18S-rRNA1/18S-rRNA2) resulted in a 0.44–1.28 kb fragment corresponding to two types of 18S rRNA genes for different *V*. *longisporum* isolates (Fig. 2b). Both types differ in an intron of 839 nucleotides, which is present in all 9 A1 rDNA *V*. *longisporum* isolates (Vl18, Vl40, Vl43, Vl59, Vl60, Bob70, Vl82, Vl83, Vl84). The intron is absent in the 18S rRNA genes of the two A1xD3 *V*. *longisporum* isolates (Vl19, Vl32) carrying the *V*. *dahliae* D3 rDNA type and is also absent in all analyzed *V*. *dahliae* and *V*. *albo*-*atrum* isolates. The 839-bp intron is therefore typical for the A1 lineage but only conserved within the virulent *V*. *longisporum* A1xD1 hybrids which have lost the D1 rDNA (Fig. 2b). A1 might have acquired this intron by horizontal gene transfer provided by another organism like a host plant (Karapapa and Typas 2001).

Our results demonstrate that the analyzed *V*. *longisporum* hybrids A1xD3 and A1xD1 which could be isolated from the same geographical region in northern Germany (Mecklenburg–Vorpommern) can be separated by their rDNA types. Only the two rDNA types A1 and D3 were maintained, the D1 rDNA type has been lost in all isolates. The virulent A1xD1 hybrids carried only the A1 rDNA including a specific intron of unknown origin which can be used to discriminate these strains from the avirulent A1xD3 hybrids carrying exclusively the D3 rDNA type which resembles the rDNA of the *V*. *dahliae* isolates.

### Both rDNA types of *V. longisporum* carry a pair of isogenes for putative regulators, which are conserved in ascomycetes

The molecular analysis of single nuclear genes is yet limited in *V*. *longisporum* and there is hardly any comparison of regulatory genes between the different *Verticillium* isolates corresponding to A1xD1, A1xD3 hybrids and related haploid species. Two orthologues of the *velvet* genes *VeA* and *VelB* were examined, which are conserved in ascomycetes. For several fungi, these regulators are known to control secondary metabolism and fungal development including hyphal morphogenesis or the formation of the conidiophore producing asexual spores (Bayram et al. 2008; Calvo 2008; Bayram and Braus 2012).

The *VEL1*-F/*VEL1*-R primers allowed only the amplification of a 1.718-kb fragment of *VeA* orthologues (*VEL1*) from genomic DNA of all nine *V*. *dahliae* isolates as well as from all 11 *V*. *longisporum* isolates (A1xD1 and A1xD3), but not from the four *V*. *albo*-*atrum* isolates. DNA sequencing resulted in the internal primer set *VEL1*-F/*VEL1*-612-R which amplified a 612-bp fragment of all isolates. The 612-bp products were cloned and sequenced and Fig. S2a summarizes the data in a phylogenetic tree. *V*. *longisporum* A1xD1 and A1xD3 carry pairs of isogenes for *VEL1* (*VlVEL1*-*1*, *VlVEL1*-*2*). The *VlVEL1*-*1* sequence of A1xD3 lineage (Vl19, Vl32) corresponds to parental D3 and is identical to *V*. *dahliae VdVEL1*. The D1 derivative *VlVEL1*-*1* of A1xD1 hybrids (Vl40, Vl43, Bob70) has accumulated three nucleotide polymorphisms (SNPs). Both lineages of *V*. *longisporum* share the same *VlVEL1*-*2* sequence which corresponds to the A1 parent and displays a 95 % identity to the single genes of *V*. *dahliae VdVEL1* or *V*. *albo*-*atrum VaVEL1* (Fig. S2a).

**Fig. 3:**
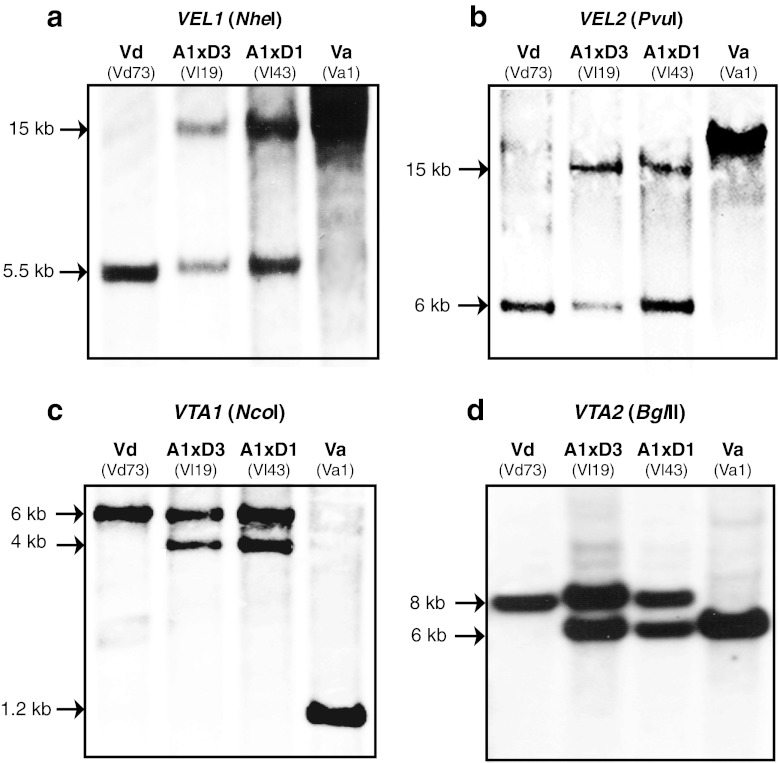
Comparative Southern hybridization for four single genes of *V*. *dahliae* (Vd) and *V*. *albo*-*atrum* (Va) in comparison to two isogenes of two *V*. *longisporum* (Vl) strains with different rDNA. **a** Genomic DNA of all representative strains was digested with *Nhe*I and probed with labeled *VEL1* DNA. The same genomic DNAs were also treated with *Pvu*I and hybridized to a *VEL2* probe (**b**), digested with *Nco*I with *VTA1* probe (**c**), and digested with *Bgl*II and probed with labeled *VTA2* (**d**)

Southern hybridization verified the two paralogues of *V*. *longisporum* and allowed a comparison of the environment of the two gene loci within the genome with the related haploid strains of *V*. *dahliae* and *V*. *albo*-*atrum* (Fig. 3a). The Southern hybridization pattern of isolates representing both hybrids (A1xD1 and A1xD3) with the different rDNA types were indistinguishable and confirmed the presence of two isogene copies in contrast to the single copies of *V*. *albo*-*atrum* or *V*. *dahliae*.

Amplification of the *VelB* homologues using the primers *VEL2*-F/*VEL2*-R (Table 2) resulted in 1.615-1.618-kb fragments. Sequencing revealed two sequences (*VlVEL2*-*1*, *VlVEL2*-*2*) for *VEL2* in *V*. *longisporum* isolates but only one in *V*. *dahliae* (*VdVEL2*) and *V*. *albo*-*atrum* (*VaVEL2*). The data are summarized in the phylogenetic tree of the Fig. S2b. *VlVEL2*-*1* of A1xD3 corresponds to the D3 parent and is identical to *V*. *dahliae VEL1* (*VdVEL1*). The corresponding D1 *VlVEL2*-*1* allele of A1xD1 carries differences in some single nucleotides. Interestingly, the *VEL2* sequences of *V*. *dahliae* Vd73 (from linseed) can be separated from other *V*. *dahliae* strains including the D3 parent of *V*. *longisporum* for *VEL2* but not for *VEL1*. Similarly, *V*. *dahliae* isolate Vd14.01 (from pistachio) has accumulated even more SNPs which separates it even more from the other *V*. *dahliae* strains than the D1 parent of the *V*. *longisporum* A1xD1 hybrid for *VEL2* but not for *VEL1* (Fig. S2b). These SNP accumulations for *VEL2* might reflect a specific yet unknown function of the *VEL2* encoded regulator for host adaptation. The other *V*. *longisporum VlVEL2*-*2* isoallele corresponds to A1 and is identical in both lineages of *V*. *longisporum* with an identity of about 95 % to both *V*. *dahliae* and *V*. *albo*-*atrum* genes.

These data suggest that similarly to *VEL1*, there are also two isogenes for *VEL2* in the *V*. *longisporum* genome. Comparison of the Southern hybridization pattern of both rDNA types revealed two loci for *VEL2* in *V*. *longisporum* including one locus which is similar to *V*. *dahliae*. The other Southern band is identical in both *V*. *longisporum* rDNA types but distinct to both *V*. *dahliae* and *V*. *albo*-*atrum* and presumably reflects the second parent A1 as unknown species which is grouped closer to *V*. *albo*-*atrum* than to the other parental strain (Fig. 3b). This further corroborates one common ancestor parent for both types of rDNA of *V*. *longisporum*.

Conclusively, our data demonstrate that in contrast to the rDNA repetitive genes, which are uniform, there are two isogenes for the putative regulators *VEL1* and *VEL2* in both *V*. *longisporum* lineages (A1xD1, A1xD3) with high identities (94–100 %) to each other and to the corresponding single genes of *V*. *dahliae* or *V*. *albo*-*atrum* as relatives to D1, D3, or A1. The reason why *V*. *dahliae* strains from specific hosts have accumulated more mutations in the *VEL2* gene remains to be elucidated.

### Paralogue transcription factor isogene pairs of *V. longisporum*

We extended our analysis by examining two orthologues of transcriptional regulatory genes, which are expressed in *V*. *longisporum*. Five thousand expressed sequence tags were sequenced from a cDNA library of *V*. *longisporum* (Singh et al. 2010). This resulted in the identification of transcripts for genes for the putative transcription factors *Verticillium* transcription activator (*VTA*)*1* and *VTA2*. The corresponding genes encoding zinc finger family proteins which are conserved within filamentous ascomycetes (Fig. S3) were cloned and sequenced.

**Fig. 4:**
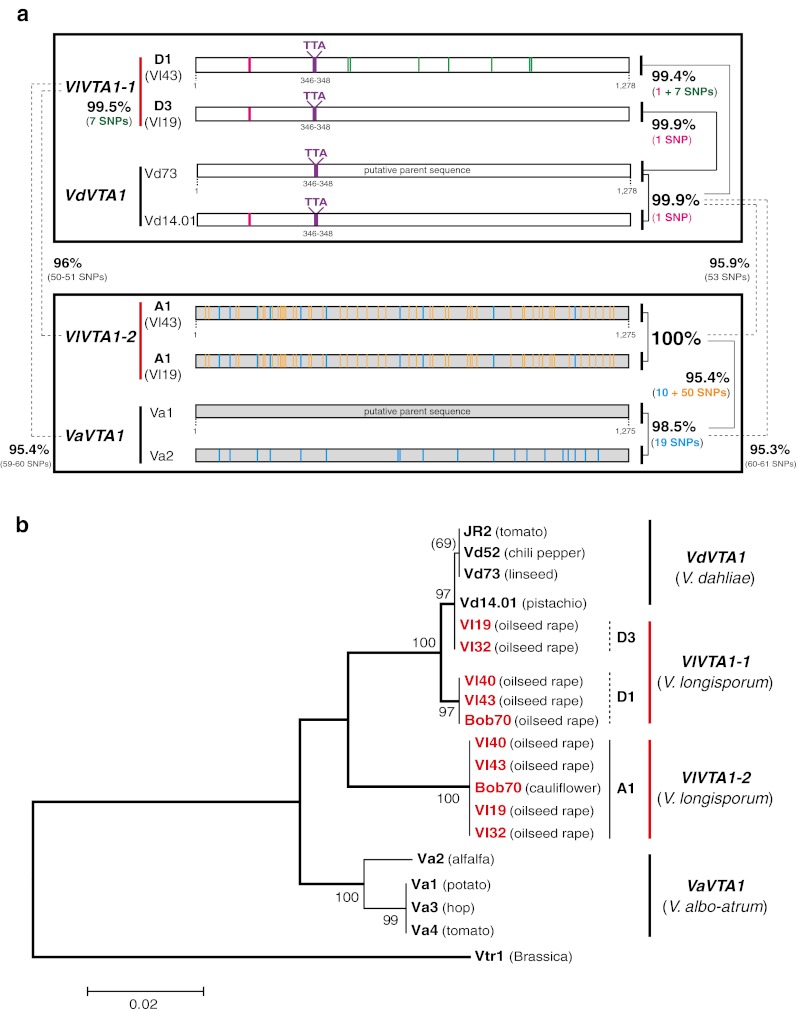
Two *VTA1* isogenes of *V*. *longisporum* (*VTA1*-*1*, *VTA1*-*2*) in comparison to the corresponding single genes of *V*. *dahliae* and *V*. *albo*-*atrum*. **a** Comparative scheme of *VTA1* from all three *Verticillium* species: Both the virulent A1xD1 isolates (Vl40, Vl43, Bob70) and the avirulent A1xD3 (Vl19, Vl32) carry two isogenes for *VTA1* gene (*VlVTA1*-*1*, *VlVTA1*-*2*), whereas only one sequence of this gene is present in *V*. *dahliae* (*VdVTA1*) and *V*. *albo*-*atrum* (*VaVTA1*). The sequences for *VlVTA1*-*1* of A1xD3 and A1xD1 are almost identical to *V*. *dahliae VdVTA1* with a minor difference in one or eight SNPs, respectively. Both *VlVTA1*-*1* and *VdVTA1* possess the same signature consisting of a three-nucleotide insertion (TTA) in the coding region. In contrast, *VlVTA1*-*2* is identical in both A1xD1 and A1xD3 and lacks the insertion of three nucleotides like *V*. *albo*-*atrum VaVTA1*. *VlVTA1*-*2* shows 95–96 % identitiy to *VdVTA2* and *VaVTA2*. Colored lines within the genes correspond to corresponding SNPs. Additional SNPs present in genes of the lower in comparison to the upper box are summarized next to the boxes and visualized by grey color. The *VdVTA1* (from Vd73 isolate) and *VaVTA1* (from Va1 isolate) are used as putative parent sequences for SNPs calculation. **b** Phylogenetic analysis based on the conserved *VTA1* gene shows close connection of *V*. *longisporum* hybrids to *V*. *dahliae* and *V*. *albo*-*atrum*

The *VTA1* gene encodes a protein containing the Zn(II)_2_Cys_6_ conserved domain. This domain is similar to the conserved domain of the AflR transcription factor in *Aspergilli* required for mycotoxin biosynthesis of the cancerogenic aflatoxin (Yu et al. 1996). The regulator Fsr6 in *Fusarium fujikuroi*, an orthologue of Vta1 (Fig. S3a), has been recently described for its function in regulation of pigment biosynthesis (Studt et al. 2012). *V*. *longisporum* carries two isogenes for *VTA1* which are both transcribed resulting in expressed sequence tags without introns. They correspond to single genes in *V*. *dahliae* or *V*. *albo*-*atrum*. The two *VTA1* sequences of 1.275 and 1.278 kb display a 95–99.9 % identity to each other and to the corresponding genes of the haploid *Verticillia* with a significant different signature: a three-nucleotide sequence in the longer *VTA1* sequence of all *V*. *longisporum* isolates (*VlVTA1*-*1*) is only present in *V*. *dahliae*. These *VlVTA1*-*1* sequences of the two *V*. *longisporum* hybrids A1xD3 (Vl19, Vl32) and A1xD1 (Vl40, Vl43, Bob70) are almost identical to *V*. *dahliae VdVTA1* with differences in one (D3) or eight (D1) single nucleotide substitutions of the 1.278-bp sequences, respectively. The *V*. *albo*-*atrum* as well as all shorter A1 *VTA1* sequences of *V*. *longisporum* (*VlVTA1*-*2*) are identical in both lineages A1xD1 and A1xD3 and include the small three-nucleotide deletion in comparison to the isoallele (Figs. 4 and S4). Consistently, comparison of the Southern hybridization pattern revealed two loci for the *VTA1* genes in both rDNA types of *V*. *longisporum*, where one locus results in the same pattern as *V*. *dahliae*, whereas there is a different pattern for the A1-derived gene which carries the same deletion as the *V*. *albo*-*atrum* gene (Fig. 3c). This further supports that A1 as the second parent species separates from its relative *V*. *albo*-*atrum* by several additional single nucleotide polymorphisms.

**Fig. 5:**
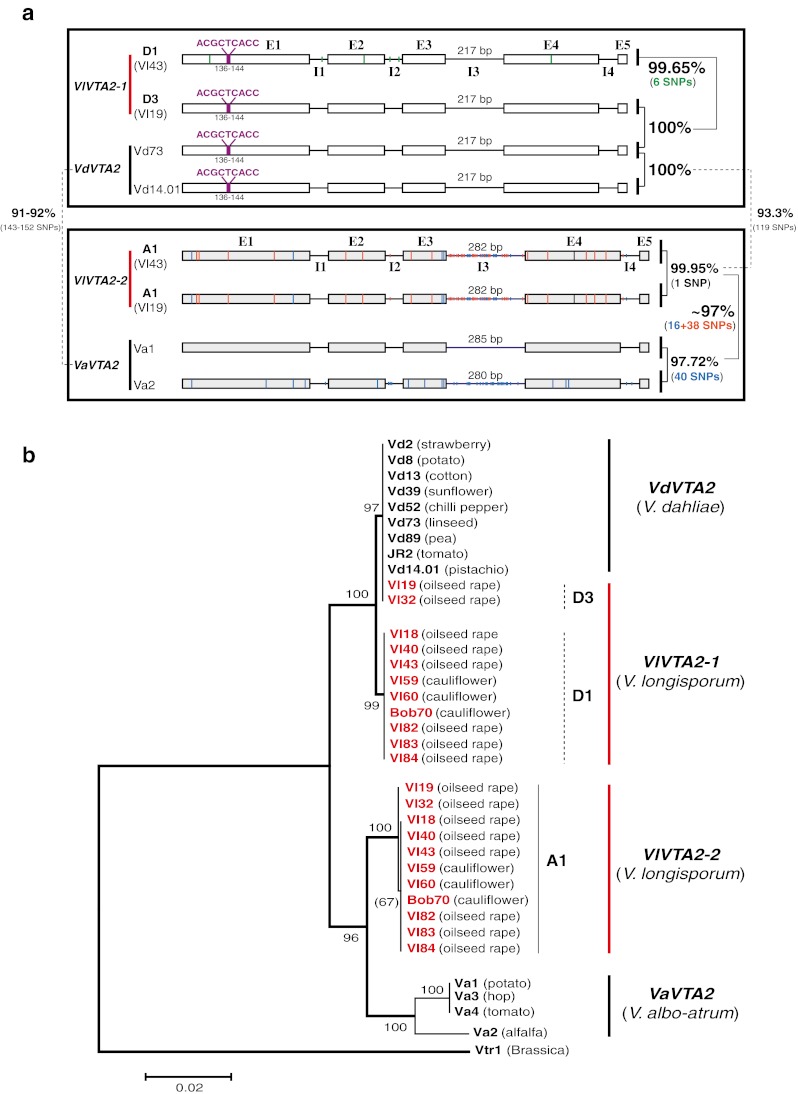
The evolutionary origin of all 11 *V. longisporum* strains based on the nuclear gene *VTA2* with the length of 1,701–1,759 nucleotides. **a** Comparative scheme of *VTA2* from all three *Verticillium* species: *VlVTA2*-*1* and *VlVTA2*-*2* isogenes of both *V*. *longisporum* lineages A1xD1 and A1xD3 are derivatives from *V*. *dahliae* and *V*. *albo*-*atrum*, respectively. Characteristic signatures include a nine-nucleotide insertion (ACGCTCACC) in the first exon (E1) and the length of the third intron (I3). *VlVTA2*-*1* and the *V*. *dahliae VdVTA2* carries the nine-nucleotide insertion in the first exon and a shortened third intron (217 bp), whereas *VlVTA2*-*2* and *V*. *albo*-*atrum VaVTA2* lacks this insertion in the first exon and carries an extended third intron (280–285 bp). *VlVTA2*-*1* of the avirulent A1xD3 isolates is identical to *V*. *dahliae VdVTA2* and differs from *VlVTA2*-*1* of the virulent A1xD1 isolates by 6 single nucleotide polymorphisms (SNPs). *VlVTA2*-*2* is identical in all A1xD1 isolates but differs from *VlVTA2*-*2* of A1xD3 by only a single nucleotide. *VlVTA2*-*2* displays 97 % identity to *V*. *albo*-*atrum VaVTA2* and only 93 % to *V*. *dahliae VdVTA2*. SNPs are indicated by colors. The *VdVTA2* (from Vd73 isolate) and *VaVTA2* (from Va1 isolate) are used as putative parent sequences for SNPs calculation. **b** Phylogenetic analysis for all 11 *V*. *longisporum* isolates in comparison to *V*. *dahliae* and *V*. *albo*-*atrum* isolates. The *VTA2* sequence of *V*. *tricorpus* is used as root of the phylogenetic tree

The *VTA2* gene containing a C_2_H_2_ conserved motif is an orthologue of the *CON7* gene in the rice blast fungus *Magnaporthe grisea*. The Con7 protein controls approximately 100 genes and is essential for appressorium formation and pathogenicity (Odenbach et al. 2007). *VTA2* genes are broadly conserved within filamentous fungi and the *Verticillium* isogenes are related to fungal plant pathogens such as *Fusarium* species and *M. grisea* (Fig. S3b). Comparison of the expressed sequence tags to the genomic sequences amplified by PCR revealed a complex gene structure for *VTA2*, which includes four introns and five exons. The *V*. *dahliae* and *V*. *albo*-*atrum VTA2* orthologues vary slightly in size with 1.701 kb and 1.754–1.759, respectively. The first exon of *V*. *dahliae VTA2* has an insertion of nine nucleotides (ACGCTCACC) when compared with *V*. *albo*-*atrum VTA2*. In addition, the third intron (I3) of the *V*. *dahliae* gene (217 bp) is 63–68 nucleotides shorter than *V*. *albo*-*atrum* I3 (280–285 bp). In contrast to the single parental genes, both types of *V*. *longisporum* rDNA carry two paralogues (Figs. 5 and S5).

The two *VTA2* isogenes for *V*. *longisporum* (*VlVTA2*-*1*, *VlVTA2*-*2*) varied in lengths slightly between 1.701 and 1.755 kb. The *VlVTA2*-*1* sequences from the two *V*. *longisporum* lineages A1xD1 and A1xD3 carry the same characteristic ACTCTCACC insertion in the first exon and the shortened third intron of 217 bp as *V*. *dahliae VTA2* gene. The *V*. *dahliae VTA2* gene is absolutely conserved in this species. *VlVTA2*-*1* of two *V*. *longisporum* A1xD3 isolates (Vl19, Vl32) carrying *V*. *dahliae* rDNA corresponds to D3 because it is identical to *VdVTA2*. This allele differs from the D1 allele of *VlVTA2*-*1* of the A1xD1 isolates carrying *V*. *albo*-*atrum*-related rDNA by six single nucleotides. In contrast, the A1 allele *VlVTA2*-*2* of the A1xD1 lineage including six European isolates (Vl18, Vl40, Vl43, Vl82, Vl83, Vl84) and three American isolates (Vl59, Vl60, Bob70) is identical and differs from the other A1 allele of *VlVTA2*-*2* of the A1xD3 lineage (Vl19, Vl32) by only a single nucleotide. This *VlVTA2*-*2* sequence exhibits an identity of about 97 % to *V*. *albo*-*atrum VaVTA2* gene. It carries the characteristic first exon of *V*. *albo*-*atrum* lacking the nine nucleotide insertion as well as the longer third intron of 282 bp (Figs. 5 and S5). Southern hybridization confirmed that the two *V*. *longisporum VTA2* isogenes are either closer related to *V*. *dahliae* or to *V*. *albo*-*atrum*, respectively (Fig. 3d).

The *VTA2* gene analyses corroborated that the two isogenes *VlVTA2*-*1* and *VlVTA2*-*2* are completely conserved within all nine *V*. *longisporum* A1xD1 isolates derived from Europe and America and have not (yet) been changed during geographical separation. Sequencing of the corresponding *VTA2* genes from nine *V*. *dahliae* isolates from different geographical regions also reveals an absolute conservation (100 % identity) of this gene. In contrast, *V*. *albo*-*atrum* isolates display minor changes in *VTA2* genes depending on hosts. The *VTA2* genes from the non-*Alfalfa* isolates (Va1, Va3, Va4) deriving from potato, hop, and tomato are completely identical to each other, but exhibit a slight reduction of 97.72 % identity compared to *VTA2* sequence of the *Alfalfa* isolate Va2. However, the *Alfalfa* and the non-*Alfalfa* group still have conserved the signatures of the first exon and the third intron (Figs. 5 and S5). This variation in nucleotide polymorphisms in *V*. *albo*-*atrum* might be the reason for the differences between the present *V*. *albo*-*atrum* isolates and the unknown *V*. *albo*-*atrum* related parental species A1 of the hybrid *V*. *longisporum*.

### *VTA2* is a barcode marker for *V. longisporum*

ITS region of nuclear rDNA as a commonly used universal DNA barcode marker for fungal taxonomy (Schoch et al. 2012) are useful to discriminate between *V*. *dahliae* and the closely related species *V*. *albo*-*atrum*. This method is limited for *V*. *longisporum*. As shown above, European *V*. *longisporum* hybrids carry either a single rDNA which corresponds to D3 *V*. *dahliae* rDNA or A1 rDNA which is closer to *V*. *albo*-*atrum* than to *V*. *dahliae* (Fig. 1). Hybrids with the D1 rDNA have not yet been described.

We searched for a simple molecular marker to discriminate between haploid and hybrid *Verticillia* by polymerase chain reaction. We initially tested two primer pairs for glyceraldehyde-3-P-dehydrogenase and mitochondrial oxalacetate transport protein genes that were reported to be specific for separation between A1xD1 and A1xD3 lineages (Inderbitzin et al. 2011). Using our strain collection, we found that both of these primer pairs do not always separate A1xD3 isolates from *V*. *dahliae*, although it is required to perform three different PCR reactions for each primer pair (Fig. S6). Furthermore, these primers were designed on basis of differences in some single nucleotide polymorphisms between two isogenes in the hybrids; therefore optimized PCR procedures are required to avoid unspecific amplifications of the homologous DNA fragments.

**Fig. 6:**
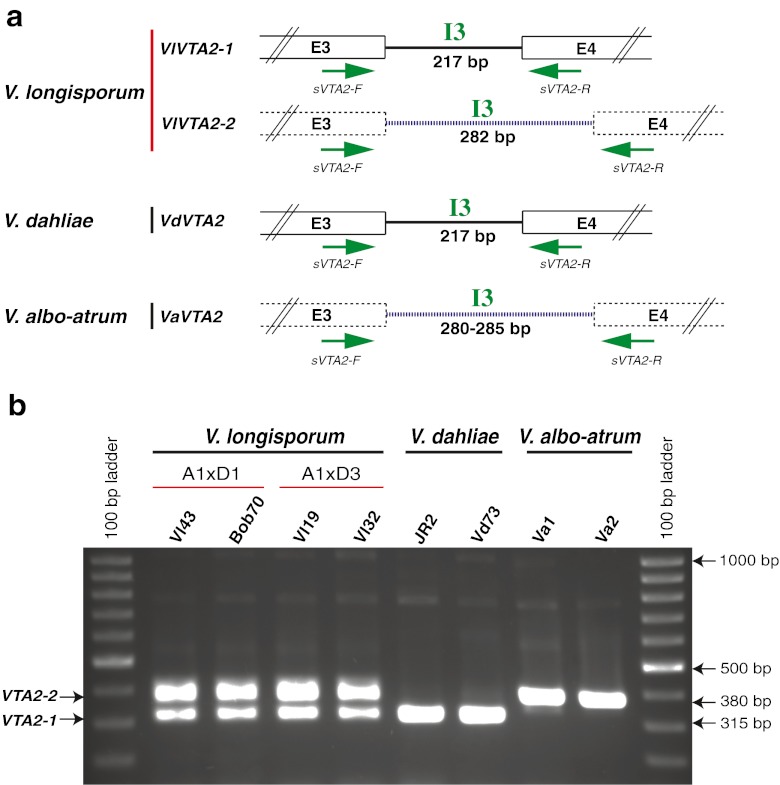
*VTA2* is a barcode gene for specific recognition of *V*. *longisporum* hybrids. **a** Scheme for the structure of the third intronic region (I3) of *VTA2* in three species. The specific primer pair (*sVTA2*-F/*sVTA2*-R) amplifying this intron is indicated by *arrows*. **b** PCR amplification with this primer pair results in two bands of 315 and 380 bp for both lineages A1xD1 and A1xD3 of the oilseed rape pathogen *V*. *longisporum*, whereas only a unique band of 315 or 380 bp is present in *V*. *dahliae* and *V*. *albo*-*atrum*, respectively

We therefore tested whether the distinct *VTA2* isogenes which are present in both lineages A1xD1 and A1xD3 of *V*. *longisporum* could overcome these disadvantages. *VlVTA2*-*1* has a similar size as *V*. *dahliae VdVTA2* (1,701 nucleotides) and *VlVTA2*-*2* resembles *V*. *albo*-*atrum VaVTA2* (1,754–1,759 nucleotides). The difference in lengths between the two *VTA2* isogenes is mainly due to differences in the third intron (Figs. 5a and 6a). We designed a specific primer pair (*sVTA2*-F/*sVTA2*-R) that amplifies only the third intron region (I3) of *VTA2* gene. These primers bind to the conserved sequences in the third (E3) and fourth exon (E4) of *VTA2* gene for all the three species (Figs. 6a and S5). With this primer pair, two bands of 315 and 380 bp, which are corresponding to *VlVTA2*-*1* and *VlVTA2*-*2*, are amplified from genomic DNA of both *V*. *longisporum* lineages A1xD1 and A1xD3, whereas only a single band of 315 or 380 bp can be amplified from genomic DNA of *V*. *dahliae* or *V*. *albo*-*atrum*, respectively (Fig. 6b).

These results show that *VTA2* can serve as barcode marker to discriminate between hybrid *V*. *longisporum* isolates and haploid *V*. *dahliae* or *V*. *albo*-*atrum* strains by a simple PCR reaction.

### Combined diagnostic tools to identify virulent A1xD1 and avirulent A1xD3 *V. longisporum* hybrids among *Verticillium* species

**Fig. 7:**
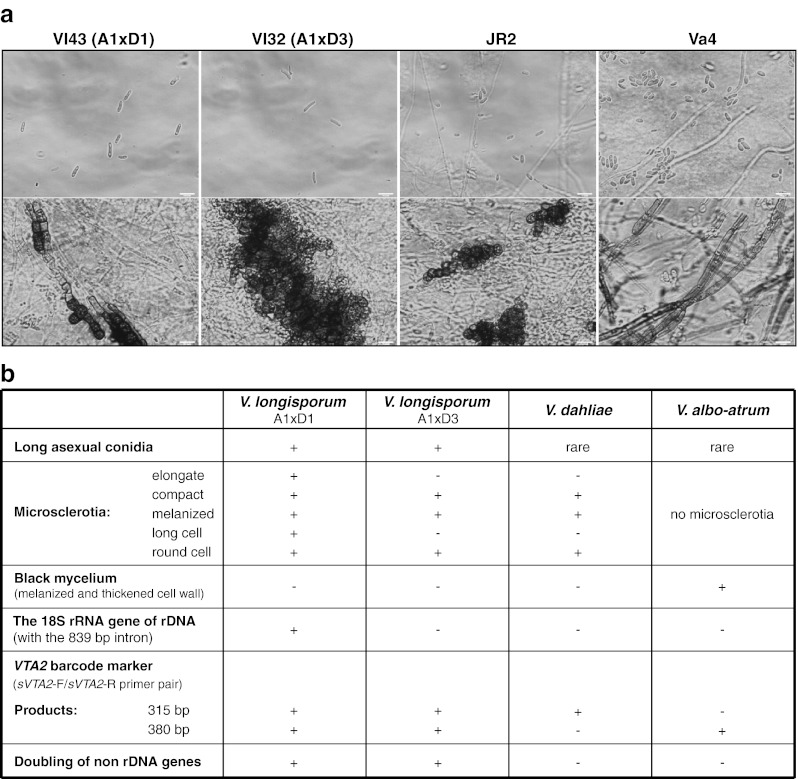
Criteria for discrimination of *V*. *longisporum* hybrids from each other and from *V*. *dahliae* and *V*. *albo*-*atrum*. **a** Morphological analysis for some representatives of *V*. *longisporum*, *V*. *dahliae* and *V*. *albo*-*atrum*. On the SXM medium mimicking xylem sap (Neumann and Dobinson 2003), two *V*. *longisporum* representative isolates produce long spores including the virulent isolate Vl43 (A1xD1) forms a mixture of elongate and compact microsclerotia whereas the avirulent one Vl32 (A1xD3) forms only compact microsclerotia comprising a group of many melanized round cells. *V*. *dahliae* produces short spores and compact microsclerotia with few melanized round cells, whereas *V*. *albo*-*atrum* also produces short spores but black hyphae as the resting structure. The same scale bars were indicated. **b** The summary of typical features as criteria for specific recognition of *V*. *longisporum* hybrids (A1xD1, A1xD3), *V*. *dahliae* and *V*. *albo*-*atrum*

The A1xD1 and A1xD3 *V*. *longisporum* hybrids from oilseed rape in Europe were originally identified by random amplified polymorphic DNA (RAPD)–PCR as groups lsp (A1xD1) or lsp* (A1xD3) which both produce longer conidia in comparison to the short spores of *V*. *dahliae* and *V*. *albo*-*atrum*. The lsp/A1xD1 group displays a high virulence on the oilseed rape *Brassica napus* by killing about 50 % plants after 42 days of infection, whereas the lsp*/A1xD3 group is significantly less virulent (Zeise and von Tiedemann 2002a, b). Like *V*. *dahliae*, both groups produce microsclerotia as resting structures. Depending on the medium, A1xD1 isolates can form elongated microsclerotia, whereas the A1xD3 isolates produce compact microsclerotia as *V*. *dahliae* (Fig. 7a). These morphological criteria vary under different environmental conditions and have to be supplemented by a set of molecular criteria (Fig. 7b). Two primer pairs specific to each isogene of the *VTA2* barcode gene and to each rDNA type for *V*. *longisporum* discriminate between virulent A1xD1 (lsp) and avirulent A1xD3 (lsp*) after analyses in morphology of conidia and the resting structures. There are two PCR bands of 315 and 380 bp for both the virulent A1xD1 and the avirulent A1xD3, but only one PCR band of 315 or 380 bp for *V*. *dahliae* and *V*. *albo*-*atrum*, respectively (Fig. 6b). In a second step, the A1xD1 isolates can be discriminated from the A1xD3 isolates by the specific primer pair for the 18S rRNA gene of rDNA. A PCR band of 1.28 kb is unique for the A1xD1 isolates, whereas the genomes of the A1xD3 isolates represent a smaller PCR band of 0.44 kb that is also present in genomes of *V*. *dahliae* and *V*. *albo*-*atrum* (Fig. 2b). This combination between the *VTA2* barcode gene analysis and the determination of the rDNA type might be a promising tool to distinguish haploid *Verticillia* from virulent A1xD1 (A1 rDNA) or avirulent A1xD3 (D3 rDNA) *V*. *longisporum* strains.

## Discussion

The two European *V*. *longisporum* hybrids A1xD1 and A1xD3 have both been isolated from oilseed rape but differ significantly in their pathogenicity towards this crop and can additionally be separated by their rDNA type. Both hybrid lineages have homogenized their rDNA to only one parental version. Two distinct rDNA types representing either the D3 *V*. *dahliae* parent or the A1 parent could be even isolated from the same site in Northern Germany. All A1xD1 *V*. *longisporum* isolates from Europe or California only represent the A1 rDNA type which is closer to *V*. *albo*-*atrum* than to *V*. *dahliae*. The D1 rDNA has not yet been found in any *V*. *longisporum* isolate. The loss of parental rDNA in *V*. *longisporum* hybrids might account for the described reduction from the diploid to the 1.8-fold ploidy of analyzed isolates. The recent classification of *V*. *longisporum* into the three different lineages A1xD1, A1xD2 and A1xD3 (Inderbitzin et al. 2011) includes the worldwide lineage A1xD1 with a broad host range including oilseed rape, cauliflower and Chinese cabbage. The A1xD2 hybrid is restricted to horseradish in Illinois, USA. A1xD3 is restricted to oilseed rape in Europe and Japan but infected plants display no disease symptoms (Zeise and von Tiedemann 2002a; Inderbitzin et al. 2011).

We analyzed here the rDNA types of A1xD1 and A1xD3 isolates and several gene pairs of putative regulators. All avirulent A1xD3 carry the D3 rDNA type; a virulent A1xD1 with a D1 rDNA was not found in the sample of 11 strains from California and Europe but all of them represented the A1 rDNA type. The analysis of several regulatory genes revealed that the alleles for strains D1 and D3 differ by several SNPs. *V*. *dahliae* strains with unusual hosts (like Vd14.01 growing on pistachio) could be more separated from other *V*. *dahliae* strains as the *V*. *longisporum* parent D1 (Fig. S2b) which has been grouped outside of *V*. *dahliae* (Inderbitzin et al. 2011).

Speciation by natural selection can favor alleles at different loci in a subpopulation in a specific environment distinct from the environment of another subpopulation. Similar selection might result in mutation-based speciation if subpopulations accumulate a different series of mutations, which is subsequently separated by ecological speciation (Schluter and Conte 2009). Fungal plant pathogens adapt rapidly to new hosts and are therefore interesting models for ecological speciation (Giraud et al. 2010). In nature, the hybrid *V*. *longisporum* infects only crops of the crucifer *Brassicaceae* family (Karapapa et al. 1997; Karapapa and Typas 2001; Zeise and von Tiedemann 2001; Barbara and Clewes 2003; Collins et al. 2003; Gladders et al. 2011), whereas *V*. *dahliae* and *V*. *albo*-*atrum* isolates infect more than 200 different plants except crucifers (Pegg and Brady 2002; Agrios 2005). The specialized inhabitation on crucifer hosts for *V*. *longisporum* might represent an ecologically dependent natural selection, where an interspecies hybrid is favored instead of its parents. At present, the hybrid can colonize ecological niches (crucifer crops) unavailable to the parents. It is currently unclear whether one parent had already accumulated some suitable specific mutations prior to hybridization, which supported the interaction to the new host or whether ecological speciation has only started after hybridization. In this study, we demonstrated that the *V*. *dahliae VEL2* gene, an orthologue of *A*. *nidulans VelB*, has an increased accumulation of SNPs in isolates from linseed (Vd73) and pistachio (Vd14.01) in comparison to the other tested *V*. *dahliae* isolates. This increased SNP accumulation might play a role in host adaptation. The VelB protein interacts with VeA and acts as a light-dependent regulator of fungal development and secondary metabolism of *A*. *nidulans* (Bayram et al. 2008; Bayram and Braus 2012). VelB orthologues are conserved in different phytopathogenic fungi for virulence. Deletion of *FfVel2* (*VelB*) in *F*. *fujikuroi* results in reduction in virulence on the host plant (Wiemann et al. 2010). Similarly, *FgVelB* deletion mutants of *Fusarium graminearum* are less virulent on wheat (Jiang et al. 2012). Vel2 (VelB) of pathogenic *Verticillium* species might also contribute to virulence on plant hosts. Accumulation of SNPs into *VEL2* gene might be involved in fitness or enhanced virulence of the pathogens on specific plants.

The ecological separation of the parental habitat disconnects gene flow between a hybrid and both parents and the speciation of the hybrid becomes more feasible (Mallet 2007). The hybrid *Zymoseptoria tritici* was shown to be the result of a hybridization event of two divergent haploid individuals. The successful spread of the wheat pathogen *Z*. *tritici* is particularly accounted to the variation in gene density and recombination rate (Stukenbrock et al. 2012). The hybrid *V*. *longisporum* lineages separated from their parental strains by specialization on cruciferous hosts. It is unclear why the A1xD1 hybrids are significantly more virulent than the A1xD3 hybrids and still can be found on the host plant *B*. *napus*.

Polyploidy increases the number of duplicated sequences in the genome resulting in homologous recombination or gene conversion mechanisms that may lead to novel intergenic interactions (Wendel 2000). Organisms with a higher ploidy level have a higher potential for distinct beneficial alleles. The rate of adaptive evolution seems to increase with the ploidy level resulting in more beneficial mutations in polyploid populations (Otto and Whitton 2000). Interspecific hybridization might start with one genome from each parent (50:50), but recombination and gene conversion may eventually lead to unequal contributions (Mallet 2007). Interspecies hybrids can lead to dominant phenotypes resulting from the combination of the parental genomes. Hybridizations between closely-related fungal species play major roles in the generation of new species to invade new host plants (Brasier et al. 1999; Staats et al. 2005). Increasing the ploidy of *V*. *longisporum* by hybridization between *V*. *dahliae* and *V*. *albo*-*atrum* related species A1 might already change the spore size. This is supported by laboratory interspecies hybridization between *V*. *dahliae* and *V*. *albo*-*atrum* (Typas 1983). Some of these artificial diploid hybrids produced long spores (8.2 ± 0.2 μm) similar to the spores of *V*. *longisporum*. It is currently unclear whether the increased spore size represents a trait, which facilitates infection of cruciferous hosts.

Polyploidy results in duplicated genes where not necessarily both genes have to remain active. Both transcription factors encoding isogene pairs that we have analyzed are presumably expressed because we found for each a corresponding cDNA derived from the transcripts. Diploidization allows that one gene could retain its original function whereas the other copy may become epigenetically silenced or inactivated by mutations. Duplicated genes might also encounter recombination, which changes the genetic locus in a concerted evolution (Wendel 2000). Physical elimination of a parental genome-specific repeated DNA occurred in wheat in amphiploids in early generations. This may play a role in the initial stabilization of the nascent amphiploid plant (Han et al. 2005). Repeated genes might homogenize and the rDNA repeats are prominent examples where one variant can become dominant via mutations. Homogenization is caused by deletion events or continuous multiplication during unequal recombination. Single nucleotide polymorphisms are present in ribosomal genes with an unexpectedly high amount (Simon and Weiss 2008). Our analysis of *V*. *longisporum* showed that homogenization of rDNA genes can shift into both directions in nature further representing an early state of species formation.

Speciation is a highly dynamic process and might end in rehaploidization. Although most of *V*. *longisporum* isolates were reported to be genetically stable (Ingram 1968; Karapapa et al. 1997; Inderbitzin et al. 2011), two natural isolates of *V*. *longisporum* from rape and sugarbeet were forced to generate haploid segregants by treatment with the haploidizing agent *p*-fluorophenylalanine. These haploid segregants produced relatively short spores and their nuclear DNA content was halved resulting in similar values as *V*. *dahliae* or *V*. *albo*-*atrum* (Jackson and Heale 1985). Haploid *Verticillium* isolates were described from crucifers as recombinant isolates or secondary haploids, which were shown to be distinct from *V*. *dahliae* as well as from *V*. *albo*-*atrum* (Karapapa et al. 1997; Collins et al. 2003). It will be interesting to monitor the future development of *V*. *longisporum* to determine the kinetics of species formation, which might correlate with a reduction of the genome size. In addition, it is still an open question if factors have changed host specificity of the hybrid. This might include changes in gene dosage of specific genes, the accumulation of specific mutations in one copy of distinct isogene pairs or combinatory effects resulting from the combination of the two parental genomes. A further reduction of the genome size might disclose these host-specificity factors, which are still hidden in the 1.8 fold genome size of *V*. *longisporum*.

The interspecies hybrid *V*. *longisporum* includes two different lineages A1xD1 and A1xD3 in Europe which both grow on *Brassicaceae* but with a significantly different impact. A1xD1 hybrids are significantly more virulent than A1xD3 hybrids. We show here that virulent A1xD1 strains have lost the D1 parental rDNA, whereas avirulent A1xD3 strains have lost the A1 rDNA. This homogenization of rDNAs allows that both *V*. *longisporum* hybrids can be distinguished by their rDNA type in a single polymerase chain reaction. The amplification of 18S rRNA with the proposed primers results in a large band (1.28 kb) for virulent A1xD1 and a small band (0.44 kb) for avirulent A1xD3. The analysis of several regulatory gene pairs in *V*. *longisporum* resulted in a second tool for classification which allows the discrimination between the hybrids and haploid *V*. *dahliae* or *V*. *albo*-*atrum* strains. The PCR examination of the barcode marker *VTA2* separates all hybrids by the resulting two bands from the single bands of *V*. *dahliae* and *V*. *albo*-*atrum*. Monitoring of the changes in the genomes of *V*. *longisporum* isolates will be an important part of a better understanding of host-specific growth of this fungus. Such diagnostic tools will support to oversee the dynamics of hybrid-mediated species formation of *Verticillia* as an emerging threat for agriculturally important crop plants.

## Electronic supplementary material

Below is the link to the electronic supplementary material.

ESM 1 PDF 15,000 kb
