# Supplementary material for: Molecular diagnosis to discriminate pathogen and apathogen species of the hybrid Verticillium longisporum on the oilseed crop Brassica napus
Source: Appl Microbiol Biotechnol. 2012 Nov 16;97(10):4467–83. doi: 10.1007/s00253-012-4530-1 (PMC3647090; doi:10.1007/s00253-012-4530-1)
Supplement: Supplementary file 1 — PDF 15,000 kb [file 253_2012_4530_MOESM1_ESM.pdf]

Electronic Supplementary Material

Applied Microbiology and Biotechnology

Molecular diagnosis to discriminate pathogen and apathogen species of the hybrid *Verticillium longisporum* on the oilseed crop *Brassica napus*

Van Tuan Tran, Susanna A. Braus-Stromeyer, Christian Timpner and Gerhard H. Braus*

Institut für Mikrobiologie und Genetik, Georg-August-Universität Göttingen, Grisebachstr. 8, D-37077 Göttingen, Germany.

***Corresponding author**: E-mail: gbraus@gwdg.de

Phone: +49-551-393771

Fax: +49-551-393330

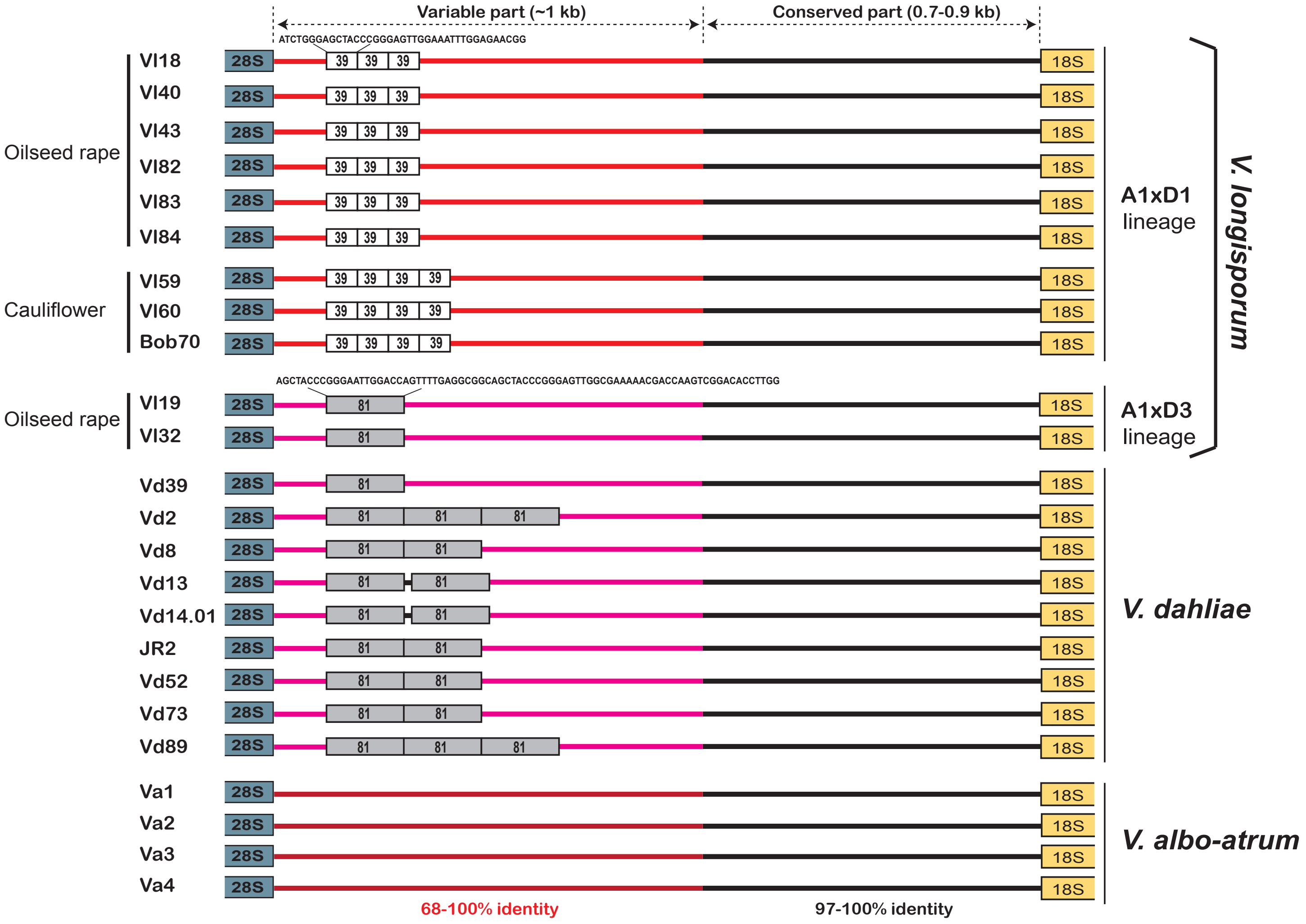

Fig. S1 Analysis of the variable region (indicated by colors) of the intergenic spacer (IGS) between rDNA genes. The A1 IGS of the virulent *V. longisporum* A1xD1 lineages includes three copies of a 39-nucleotide repeat in the IGS region from European oilseed rape and four repeat copies in the IGS of the American A1xD1 isolates from cauliflower. *V. albo-atrum* IGS differs from A1 IGS and has no specific repeats in the IGS region of rDNA. The D3 IGS of A1xD3 lineages carries a signature of 81 nucleotides that is also present in the IGS of other *V. dahliae* strains. Whereas the D3 IGS of A1xD3 hybrids includes a single copy of the 81-nucleotide signature like the IGS of Vd39 from sunflower, there are two or three repeats of this sequence in the IGS of the other analyzed *V. dahliae* isolates

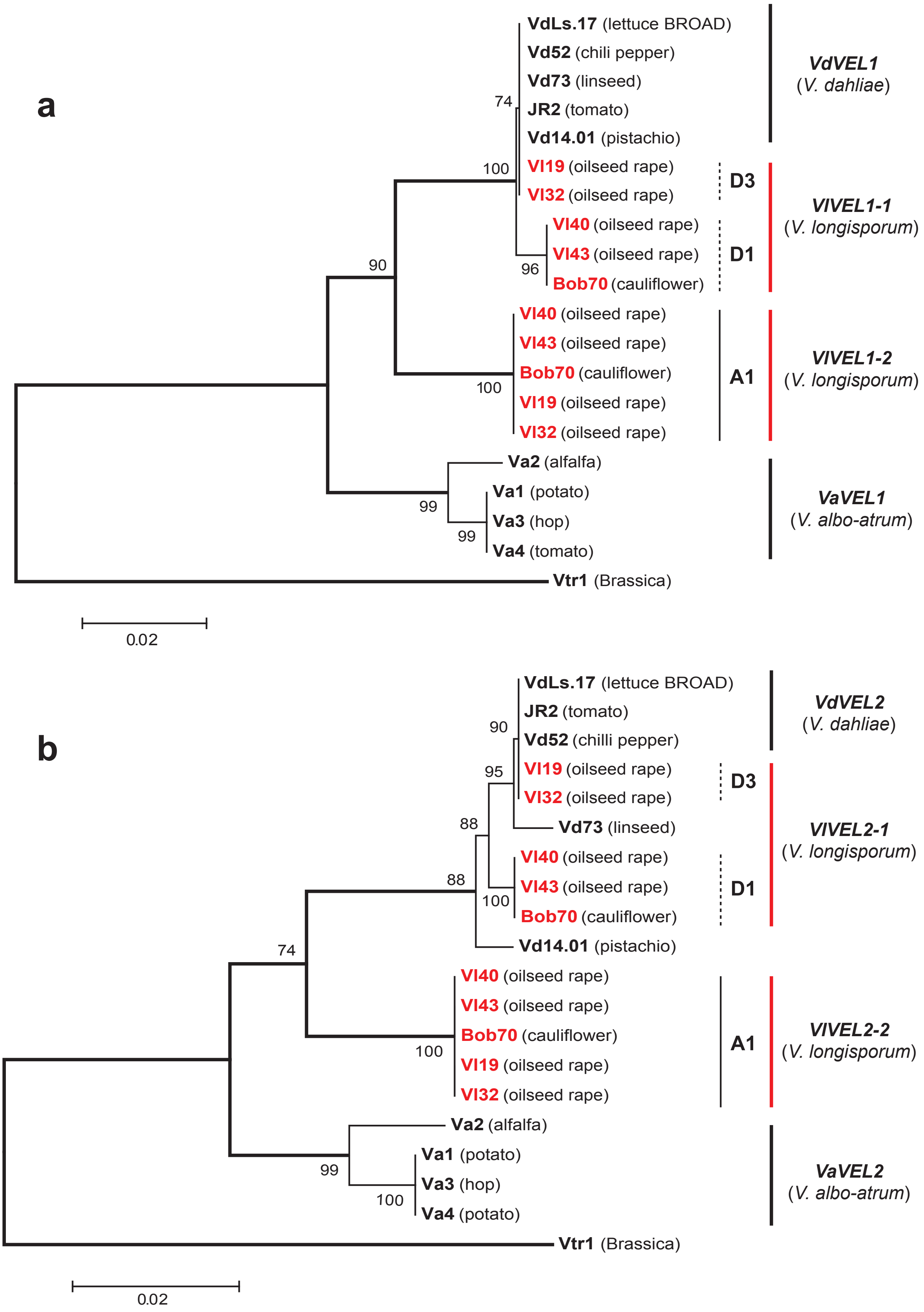

Fig. S2 The phylogenetic trees of the hybrid fungus *Verticillium longisporum* based on the (partial) sequencing of two velvet-like genes, *VEL1* (612 bp) and *VEL2* (1,615 bp). (**a**) All 11 *V. longisporum* isolates carry two isogenes (*VlVEL1-1*, *VlVEL1-2*) for *VEL1* gene, whereas only a

single sequence of this gene is present in *V. dahliae* and *V. albo-atrum*. The *VlVEL1-1* sequence of A1xD3 lineage (Vl19, Vl32) is identical to *V. dahliae VdVEL1* but distinguished from *VlVEL1-1* of A1xD1 by 3 SNPs. Both lineages of *V. longisporum* share the same *VlVEL1-2* sequence that displays a 95% identity to both *VdVEL1* and *VaVEL1*. (**b**) There are also two sequences for *VEL2* in *V. longisporum* (*VlVEL2-1*, *VlVEL2-2*) in which *VlVEL2-1* of A1xD3 is identical to *V. dahliae VEL1* (*VdVEL1*) and share 99% identity to *VlVEL2-1* of A1xD1 that might have accumulated some single nucleotide polymorphisms (SNPs) during host adaptation. The *VlVEL2-2* is identical in both lineages of *V. longisporum* and shares the identity of about 95% to both *V. dahliae* and *V. albo-atrum*. Interestingly, the *VEL2* sequences of two *V. dahliae* isolates including Vd73 (from linseed) and Vd14.01 (from pistachio) have accumulated some SNPs to be differentiated from the other *V. dahliae* isolates

a

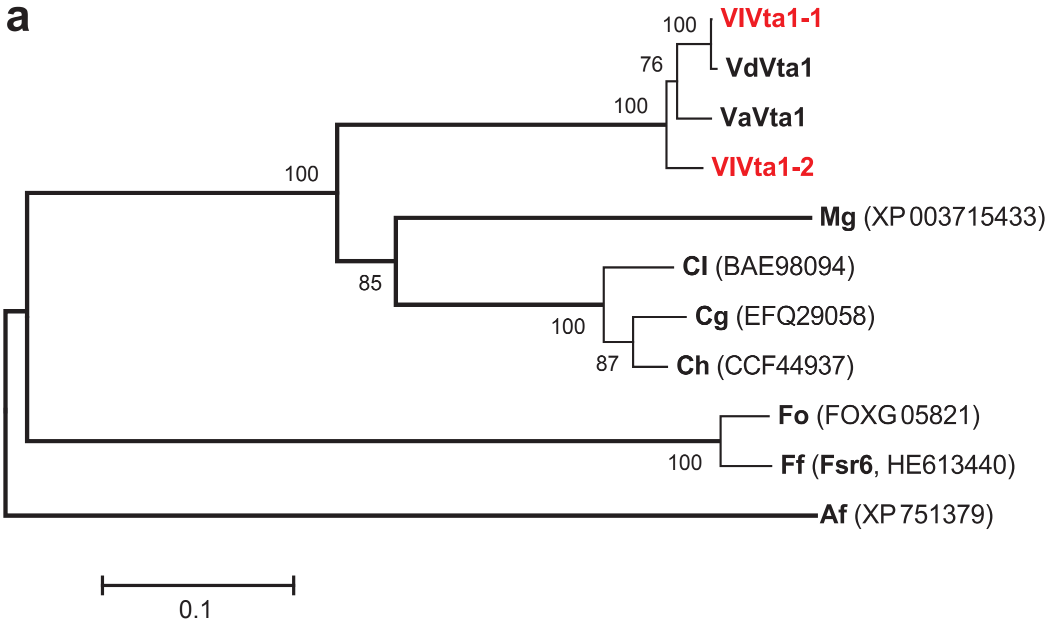

b

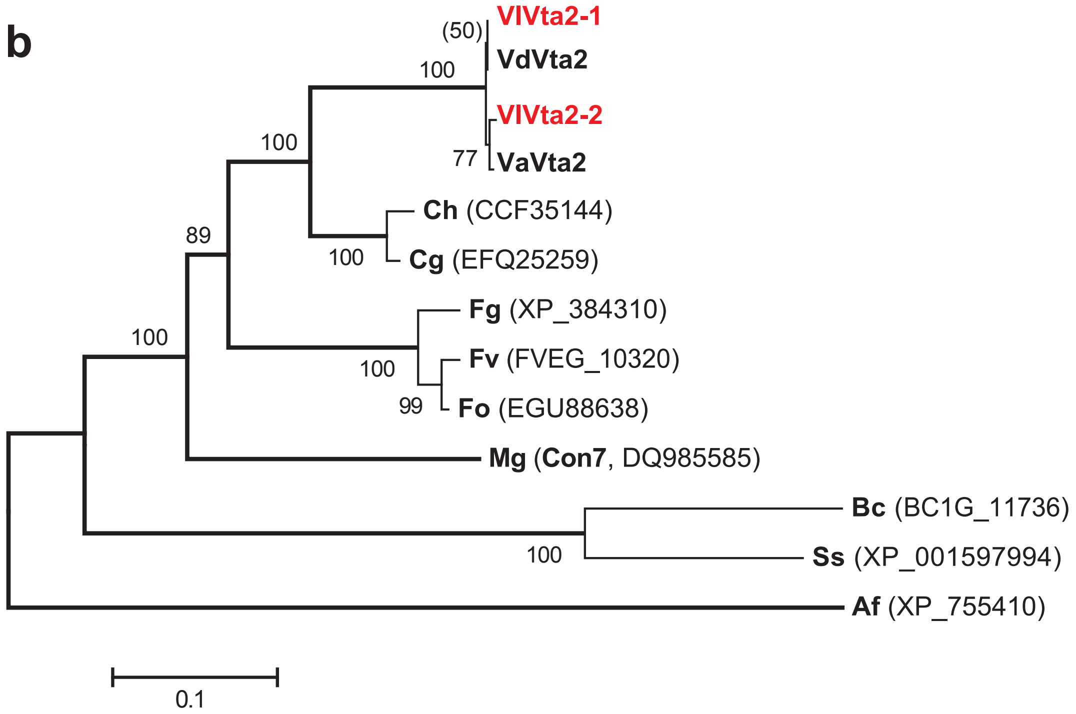

Fig. S3 Two putative transcription factors Vta1 and Vta2 are conserved in ascomycetes. Phylogenetic comparisons of Vta1 (**a**) and Vta2 (**b**) display their broad conservation in the filamentous fungi. Vta1 is a putative orthologue of *Fusarium fujikuroi* Fsr6 that controls the *PKS* gene cluster for pigmentation (Studt et al. 2012) and carries a conserved motif similar to the DNA-

binding domain of AflR that is responsible for aflatoxin biosynthesis in *Aspergillus* species (Yu et al. 1996). Vta2 is an orthologue of Con7 in *Magnaporthe grisea* that is required for virulence. Orthologues of Vta1 and Vta2 were extracted from Genbank or BROAD fungal databases (http://www.broadinstitute.org/scientific-community/science/projects/fungal-genome-initiative/fungal-genome-initiative) by using blast search. Mg = *M. grisea*, Cg = *Colletotrichum graminicola*, Cl = *C. lagenarium*, Ch = *C. higginsianum*, Fg = *F. graminearum*, Fo = *F. oxysporum*, Fv = *F. verticillioides*, Ff = *F. fujikuroi*, Bc = *Botrytis cinerea*, Ss = *Sclerotinia sclerotiorum*, Af = *A. fumigatus*

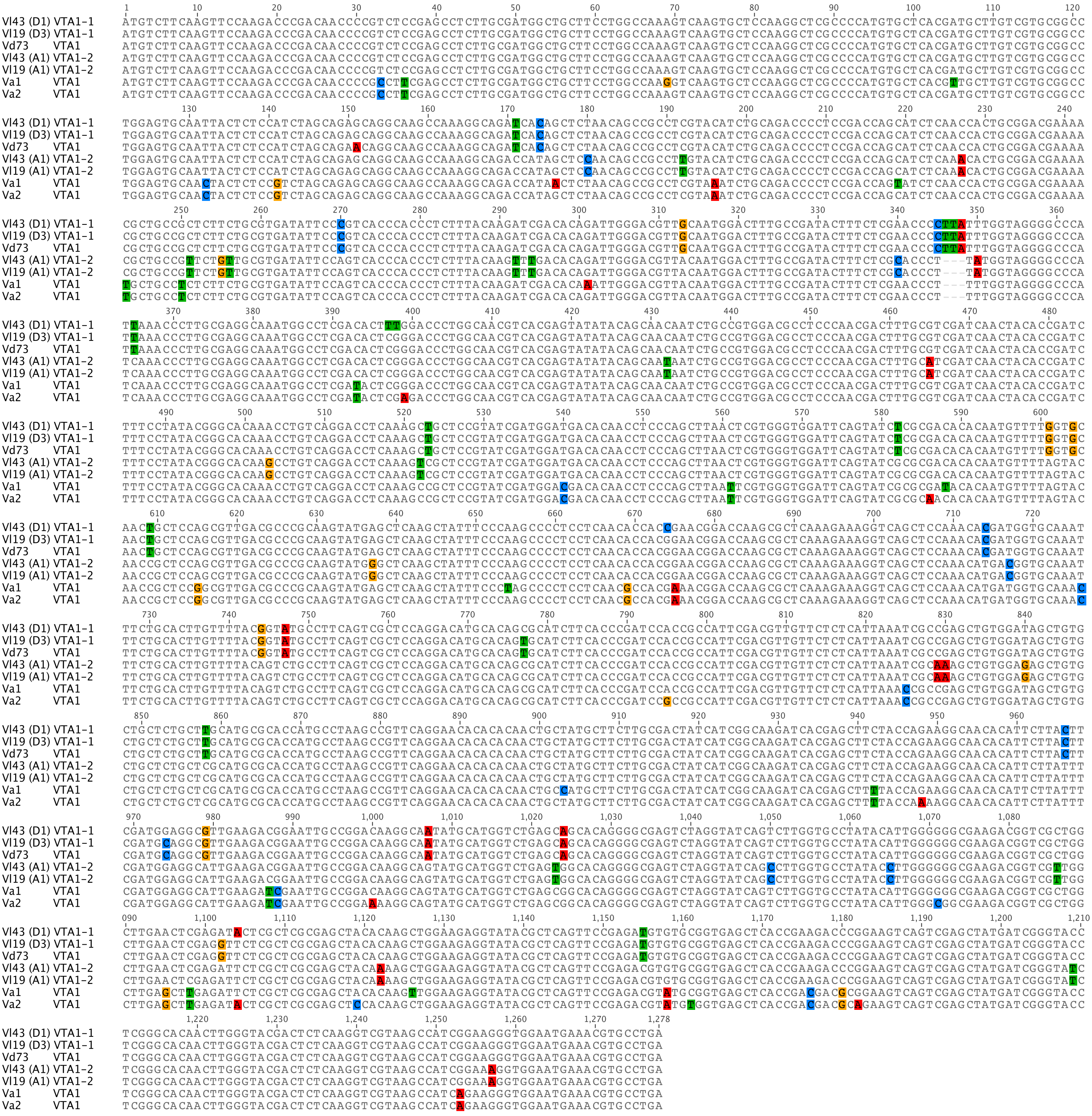

Fig. S4 Comparative alignment of the *VTA1* sequences from all three species. The alignment was performed with the commercial software Geneious Pro v5.4 (Drummond et al. 2011). The single nucleotide polymorphisms (SNPs) were indicated by colors

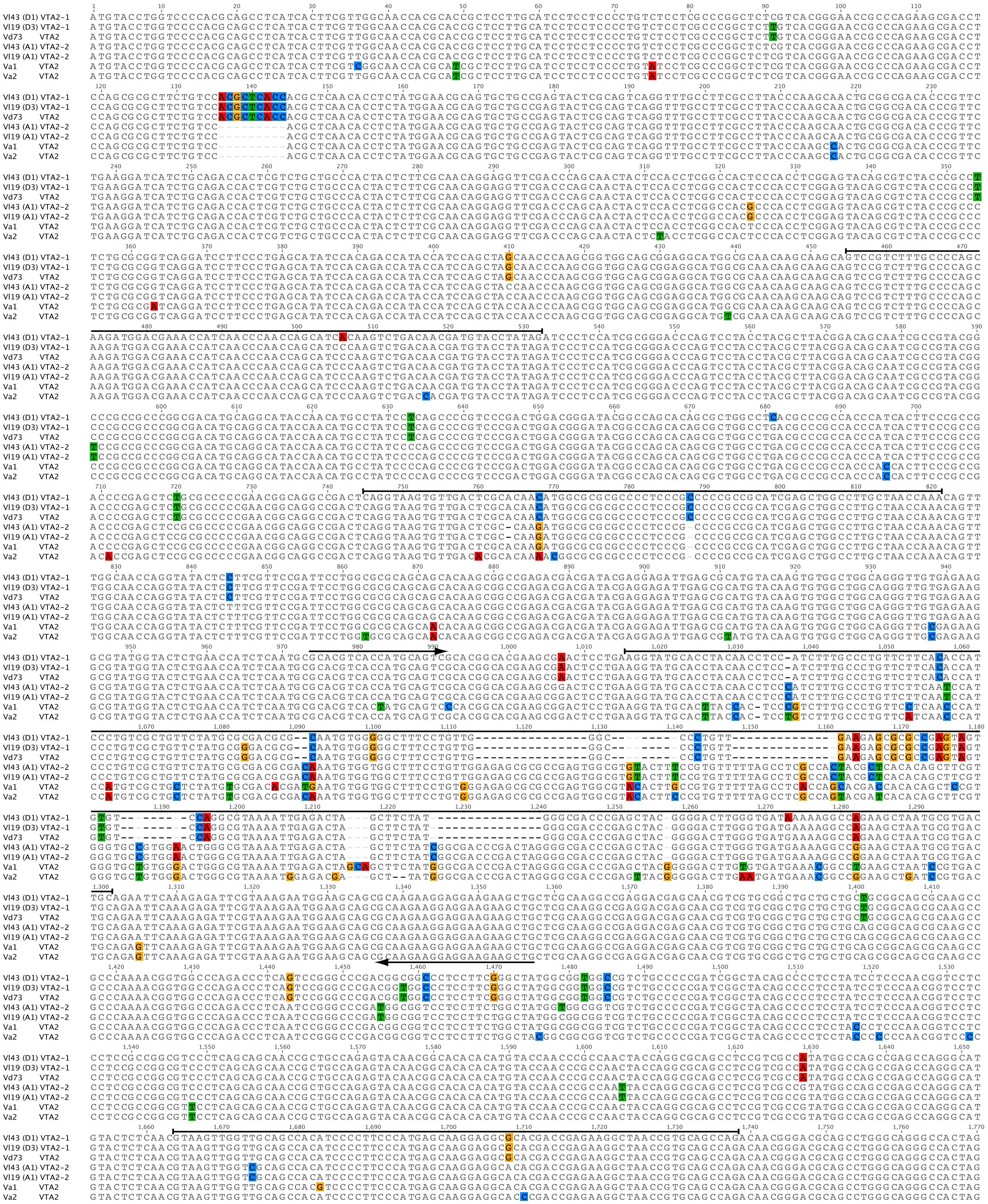

Fig. S5 Comparative alignment of the *VTA2* sequences from all three species. The differences in SNPs were shown by colors. The four introns of the gene and their sizes were indicated by restricted lines. The specific primer pair (*sVTA2*-F/*sVTA2*-R) is displayed by arrows

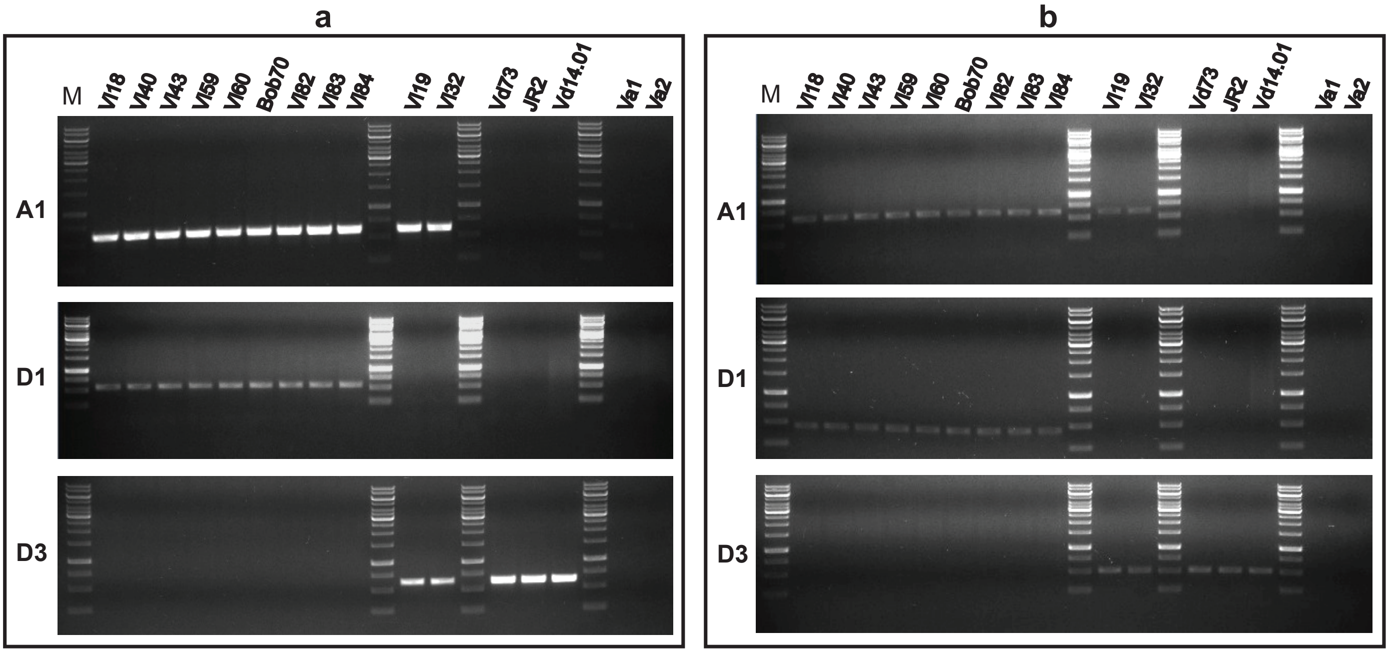

Fig. S6 PCR patterns of all 11 *V. longisporum*, 3 *V. dahliae* and 2 *V. albo-atrum* isolates using sequence-specific primers. *GPD* gene, glyceraldehyde-3-phosphate dehydrogenase (**a**) and *OX* gene, mitochondrial oxaloacetate transport protein (**b**) were amplified from the isolates using the corresponding specific primers from Inderbitzin et al. (2011). One band corresponding to the sequence of species A1 is present in all *V. longisporum* isolates, whereas the other band corresponding to the sequence of D1 or D3 is only present in the A1xD1 or in the A1xD3 isolates and the *V. dahliae* isolates
